# Supplementary material for: Multiple reaction monitoring assays for large-scale quantitation of proteins from 20 mouse organs and tissues
Source: Commun Biol. 2024 Jan 2;7:6. doi: 10.1038/s42003-023-05687-0 (PMC10762018; doi:10.1038/s42003-023-05687-0)
Supplement: Supplementary file 2 — Supplementary Information [file 42003_2023_5687_MOESM2_ESM.pdf]

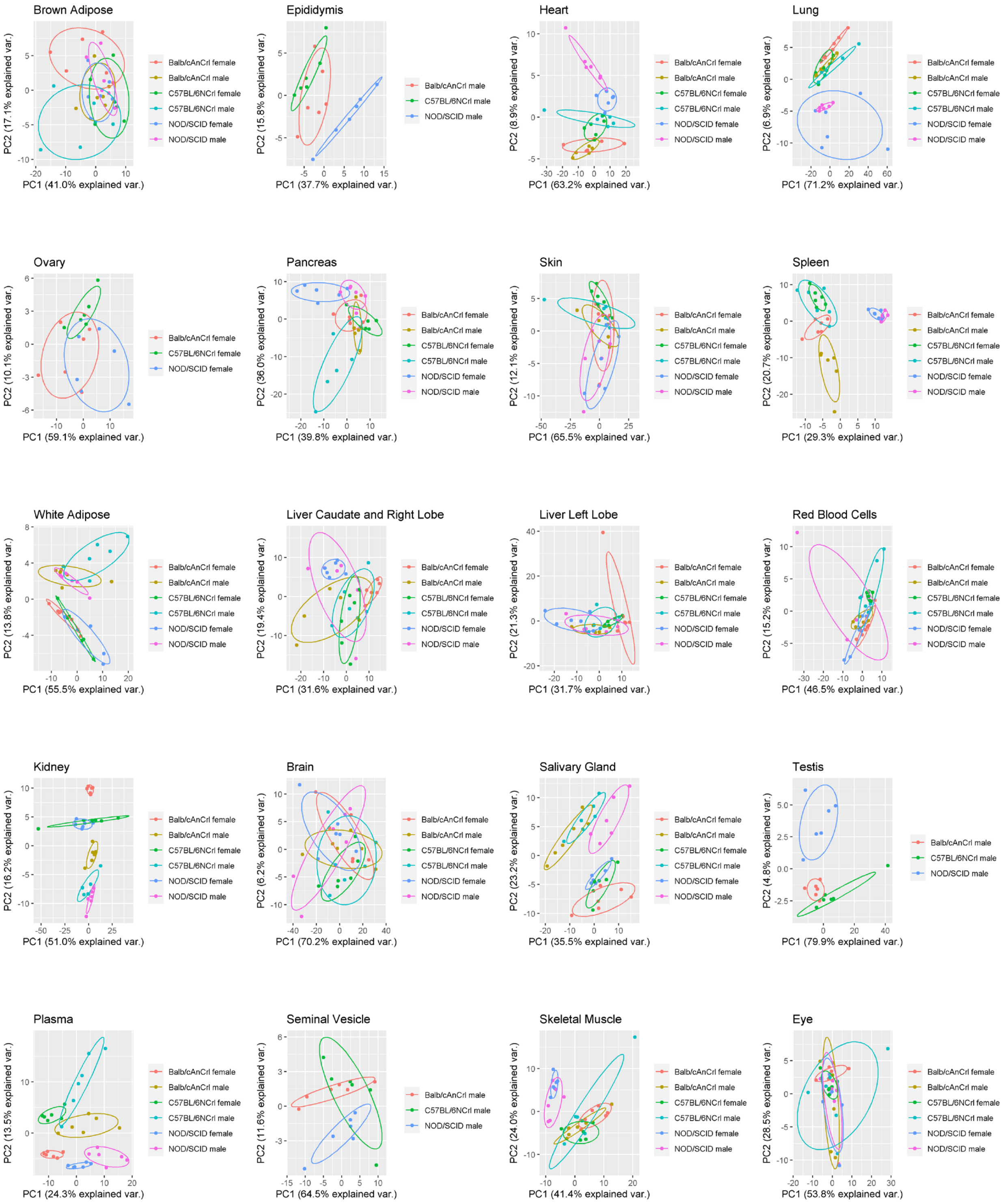

Supplementary Figure 1. PCA plots of 20 tissue and organ samples from three mouse strains. Some sample such as eye and skin showed little to no grouping according to the measured protein concentrations, while other tissues such as spleen show distinct separation between strain and/or sex.

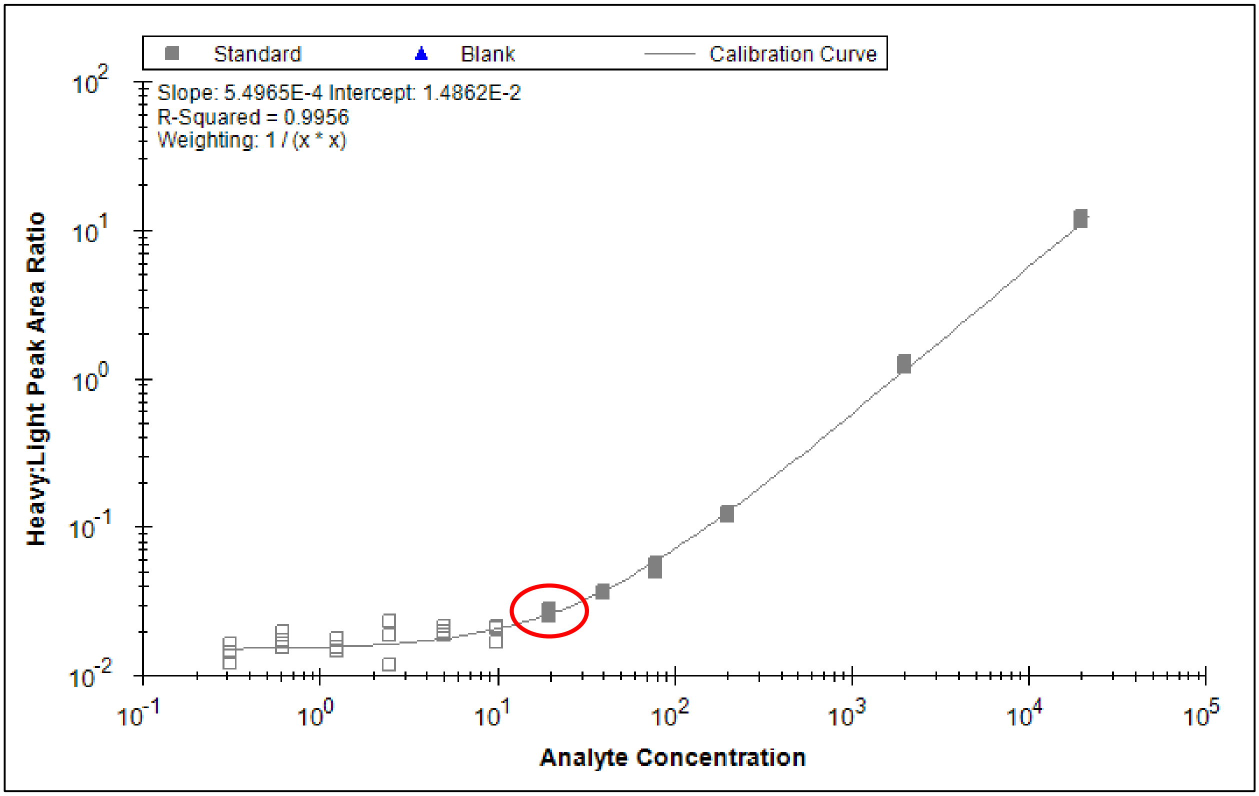

Supplementary Figure 2. Response curve for the peptide FPLIEQTYYPNHR (Synapsin-2, Q64332). Response curve spans 12 points ranging from 20,000 to 0.3125 fmol peptide/injection. Points which were excluded are depicted by unshaded squares; included points are shaded squares. The linear range was defined as the concentrations for which the mean peak area ratio was within ± 20% of the expected concentration. For this assay, the LLOQ was determined to be 20 fmol peptide/injection (circled in red), which was the lowest spiking concentration within the linear range where the coefficient of variation (CV) was less than 20%.

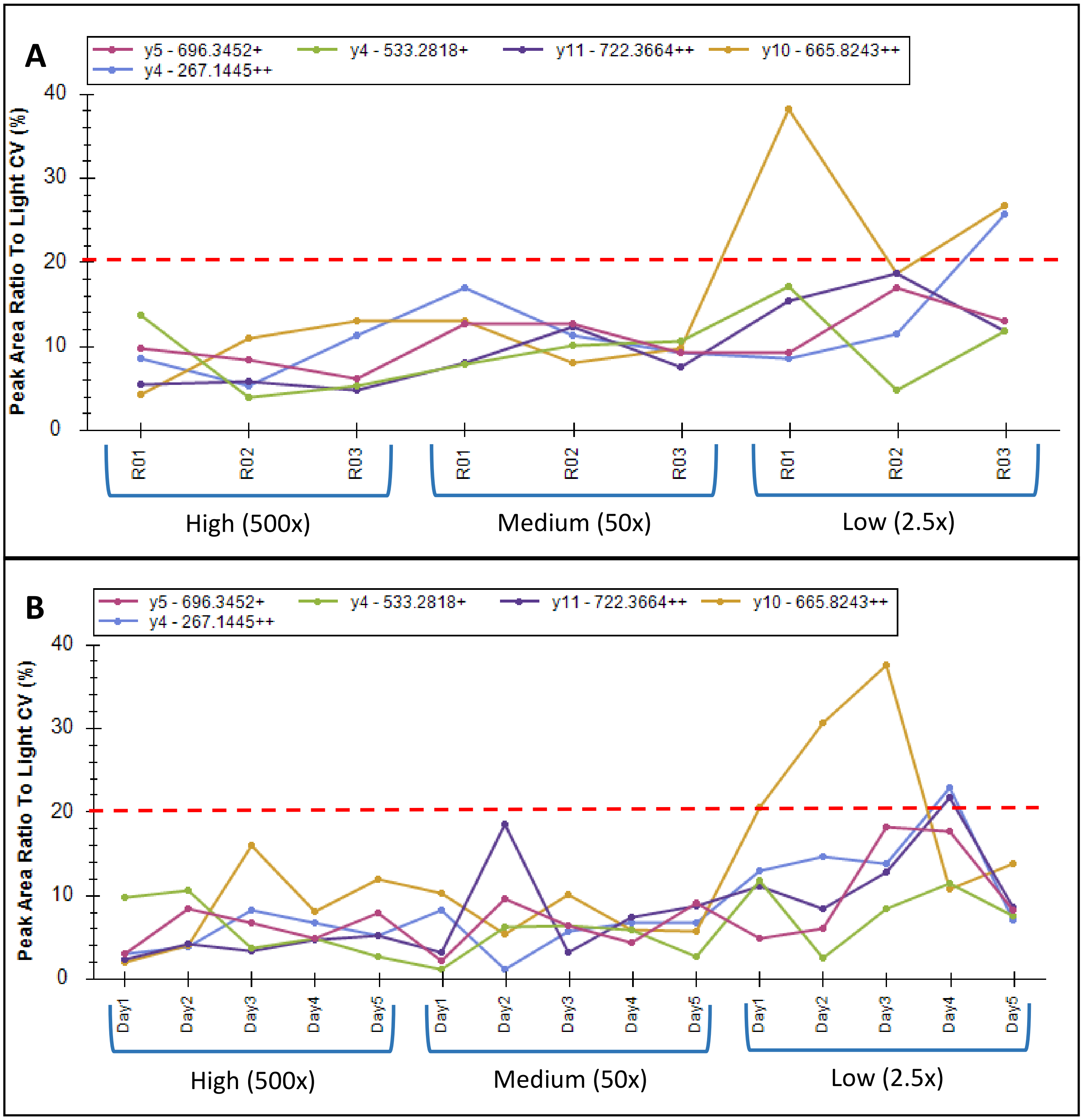

Supplementary Figure 3. Plots depicting inter-assay variability (A) and intra-assay variability (B) for the peptide FPLIEQTYYPNHR (Synapsin-2, Q64332) at three spiking concentrations relative to the assay's LLOQ: high (500x), medium (50x) and low (2.5x). Each concentration was injected in triplicate on 5 separate days (45 injections in total). Inter-assay variability (A) is calculated at each concentration as the coefficient of variation (CV) for each replicate injection across the five days; the CV values for each replicate is then averaged. Intra-assay variability is calculated at each concentration as the CV of the three replicates on each of the five days; the CV values for each day are then averaged. The total CV is calculated as the square root of the sum of (average intra-assay CV)^2 and (average inter-assay CV)^2.

Synapsin-2 FPLIEQTYYPNHR

Inter-assay Variability

	y10++			y11++			y4+			y4++			y5+		
	low	med	high	low	med	high	low	med	high	low	med	high	low	med	high
**R01**	38.2	13.0	4.3	15.3	8.0	5.3	17.0	7.8	13.7	8.4	16.9	8.5	9.1	12.6	9.7
**R02**	18.7	7.9	10.9	18.5	12.3	5.7	4.7	10.0	3.8	11.4	11.2	5.3	16.9	12.5	8.3
**R03**	26.6	9.6	12.9	11.7	7.5	4.6	11.7	10.5	5.2	25.6	9.2	11.2	12.9	9.1	6.2
**Average**	27.8	10.2	9.4	15.2	9.3	5.2	11.1	9.4	7.6	15.1	12.4	8.3	13.0	11.4	8.1

Intra-assay Variability

	y10++			y11++			y4+			y4++			y5+		
	low	med	high	low	med	high	low	med	high	low	med	high	low	med	high
**Day 1**	20.5	10.2	1.9	11.0	3.1	2.2	11.8	1.2	9.7	12.8	8.2	3.0	4.9	2.2	3.0
**Day 2**	30.6	5.4	4.0	8.4	18.4	4.2	2.5	6.1	10.5	14.5	1.1	3.8	5.9	9.6	8.4
**Day 3**	37.5	10.0	15.8	12.8	3.1	3.4	8.4	6.4	3.6	13.7	5.7	8.1	18.1	6.3	6.6
**Day 4**	10.8	5.8	7.9	21.6	7.3	4.6	11.3	5.8	4.8	22.8	6.7	6.6	17.7	4.3	4.7
**Day 5**	13.7	5.6	11.9	8.5	8.8	5.1	7.6	2.7	2.7	7.0	6.6	5.2	8.3	8.9	7.8
**Average**	22.6	7.4	8.3	12.5	8.1	3.9	8.3	4.4	6.3	14.2	5.6	5.3	11.0	6.3	6.1

Total Variability

	y10++			y11++			y4+			y4++			y5+		
	low	med	high	low	med	high	low	med	high	low	med	high	low	med	high
**Total Variability**	35.8	12.6	12.5	19.7	12.3	6.5	13.9	10.4	9.8	20.7	13.7	9.9	17.0	13.0	10.1
**Validated**	N			Y			Y			N			Y		

Supplementary Figure 4. Peak area coefficients of variation calculated for determining of inter-assay variability, intra-assay variability, and total variability for the peptide FPLIEQTYYPNHR (Synapsin-2, Q64332) at three spiking concentrations relative to the assay's LLOQ: high (500x), medium (50x) and low (2.5x).
