# Supplementary material for: Multiple reaction monitoring assays for large-scale quantitation of proteins from 20 mouse organs and tissues
Source: Commun Biol. 2024 Jan 2;7:6. doi: 10.1038/s42003-023-05687-0 (PMC10762018; doi:10.1038/s42003-023-05687-0)
Supplement: Supplementary file 3 — Description of Additional Supplementary Files [file 42003_2023_5687_MOESM3_ESM.pdf]

Description of Additional Supplementary Files

File name: Supplementary Data 1
Description: Overview of Assays - overview of peptide/protein identification, assay development, and multiplexing into organ or tissue-specific panels.

File name: Supplementary Data 2
Description: Untargeted Peptides IDs - Peptides identified by untargeted MS analysis in each sample type.

File name: Supplementary Data 3
Description: Untargeted Protein IDs - Proteins identified by untargeted MS analysis in each sample type.

File name: Supplementary Data 4
Description: Synthesized Peptides - Characteristics of all synthesized peptides.

File name: Supplementary Data 5
Description: Assay Validation - Characteristics of assays validated in each sample type.
