# Supplementary material for: Multiple reaction monitoring assays for large-scale quantitation of proteins from 20 mouse organs and tissues
Source: Commun Biol. 2024 Jan 2;7:6. doi: 10.1038/s42003-023-05687-0 (PMC10762018; doi:10.1038/s42003-023-05687-0)
Supplement: Supplementary file 5 — Reporting Summary [file 42003_2023_5687_MOESM5_ESM.pdf]

nature portfolio

Corresponding author(s): Sarah Michaud

Last updated by author(s): Nov 27, 2023

Reporting Summary

Nature Portfolio wishes to improve the reproducibility of the work that we publish. This form provides structure for consistency and transparency in reporting. For further information on Nature Portfolio policies, see our Editorial Policies and the Editorial Policy Checklist.

Statistics

For all statistical analyses, confirm that the following items are present in the figure legend, table legend, main text, or Methods section.

n/a	Confirmed	
☐	☒	The exact sample size (*n*) for each experimental group/condition, given as a discrete number and unit of measurement
☐	☒	A statement on whether measurements were taken from distinct samples or whether the same sample was measured repeatedly
☐	☒	The statistical test(s) used AND whether they are one- or two-sided<br>*Only common tests should be described solely by name; describe more complex techniques in the Methods section.*
☒	☐	A description of all covariates tested
☐	☒	A description of any assumptions or corrections, such as tests of normality and adjustment for multiple comparisons
☐	☒	A full description of the statistical parameters including central tendency (e.g. means) or other basic estimates (e.g. regression coefficient) AND variation (e.g. standard deviation) or associated estimates of uncertainty (e.g. confidence intervals)
☐	☒	For null hypothesis testing, the test statistic (e.g. *F*, *t*, *r*) with confidence intervals, effect sizes, degrees of freedom and *P* value noted<br>*Give P values as exact values whenever suitable.*
☒	☐	For Bayesian analysis, information on the choice of priors and Markov chain Monte Carlo settings
☒	☐	For hierarchical and complex designs, identification of the appropriate level for tests and full reporting of outcomes
☒	☐	Estimates of effect sizes (e.g. Cohen's *d*, Pearson's *r*), indicating how they were calculated

Our web collection on statistics for biologists contains articles on many of the points above.

Software and code

Policy information about availability of computer code

Data collection	No software was used to collect/generate the results in this study.
Data analysis	Untargeted MS data were processed using Proteome Discoverer (version 2.2.0.388) which searched the data against the mouse reference proteome (Uniprot) using the MASCOT search engine. All MRM raw data were processed and inspected using the Skyline Daily software.

For manuscripts utilizing custom algorithms or software that are central to the research but not yet described in published literature, software must be made available to editors and reviewers. We strongly encourage code deposition in a community repository (e.g. GitHub). See the Nature Portfolio guidelines for submitting code & software for further information.

Data

Policy information about availability of data

All manuscripts must include a data availability statement. This statement should provide the following information, where applicable:

- Accession codes, unique identifiers, or web links for publicly available datasets
- A description of any restrictions on data availability
- For clinical datasets or third party data, please ensure that the statement adheres to our policy

The raw data from the untargeted MS experiments have been deposited to the ProteomeXchange Consortium via the PRIDE partner repository with the dataset identifier PXD021333. A summary of the data generated during assay development are found in Supplementary Tables 4 and 5. The reference sample concentration data can be accessed via Panorama Public at https://panoramaweb.org/MRMmouse20tissues.url and at ProteomeXchange with ID number PXD020930. The protein

abundances measured in tissues from three commonly used mouse strains during this study are also hosted in an online, interactive knowledgebase, MouseQuaPro, which displays the concentration of each protein along with additional information about the protein's biological function, human orthologues, and involvement in disease.

Human research participants

Policy information about studies involving human research participants and Sex and Gender in Research.

Reporting on sex and gender	NA
Population characteristics	NA
Recruitment	NA
Ethics oversight	NA

Note that full information on the approval of the study protocol must also be provided in the manuscript.

Field-specific reporting

Please select the one below that is the best fit for your research. If you are not sure, read the appropriate sections before making your selection.

☒ Life sciences ☐ Behavioural & social sciences ☐ Ecological, evolutionary & environmental sciences

For a reference copy of the document with all sections, see nature.com/documents/nr-reporting-summary-flat.pdf

Life sciences study design

All studies must disclose on these points even when the disclosure is negative.

Sample size	Sample sizes during assay development followed the CPTAC guidelines for MRM assay reproducibility. Sample sizes for mice used in the measurement of protein concentrations were based on our previous publication (Michaud, S. A. et al. Molecular phenotyping of laboratory mouse strains using 500 multiple reaction monitoring mass spectrometry plasma assays) and increased to n = 6 male, n = 6 female mice per strain.
Data exclusions	No samples or animals were excluded from the study. The concentration data for each organ or tissue type was filtered during analysis to remove "undetectable" peptides for which 50% or more of all measurements were below one half the assay's LLOQ.
Replication	Assay development was performed according to the CPTAC guidelines to ensure appropriate replication and MRM assay reproducibility. Pooled matrix samples for the development of quantitative MRM-MS assays were prepared by combining homogenized, digested organ or tissue samples from n = 3 male and n = 3 female C57BL/6NCrl mice. Protein quantitation experiments measured individual organ or tissue samples from n = 6 male and n = 6 female C57BL/6NCrl, BALB/cAnCrl, and NOD/SCID mice (36 mice total).
Randomization	For each tissue type, samples from individual mice for concentration measurement were added to the 96 well plate in a randomized order. Sample preparation and MRM-MS acquisition were performed in this randomized order. The development and use of tissue-specific panels prevented randomization with respect to tissue type.
Blinding	Samples from individual mice for concentration measurement were assigned randomized order and each batch processed to completion regardless of mouse strain. The development and use of tissue-specific panels prevented blinding with respect to tissue type.

Reporting for specific materials, systems and methods

We require information from authors about some types of materials, experimental systems and methods used in many studies. Here, indicate whether each material, system or method listed is relevant to your study. If you are not sure if a list item applies to your research, read the appropriate section before selecting a response.

Materials & experimental systems

n/a	Involved in the study
☒	☐ Antibodies
☒	☐ Eukaryotic cell lines
☒	☐ Palaeontology and archaeology
☐	☒ Animals and other organisms
☒	☐ Clinical data
☒	☐ Dual use research of concern

Methods

n/a	Involved in the study
☒	☐ ChIP-seq
☒	☐ Flow cytometry
☒	☐ MRI-based neuroimaging

Animals and other research organisms

Policy information about studies involving animals; ARRIVE guidelines recommended for reporting animal research, and Sex and Gender in Research

Laboratory animals	Samples were collected from n = 6 male and n = 6 female 12 week old mice from three strains (C57BL/6NCrl, BALB/cAnCrl, and NOD/SCID) for protein quantitation experiments, and from n = 3 male and n = 3 female C57BL/6NCrl mice for assay development experiments.
Wild animals	The study did not involve wild animals.
Reporting on sex	Equal numbers of male and female mice were used for sample collection throughout study: Samples were collected from n = 6 male and n = 6 female 12 week old mice from three strains (C57BL/6NCrl, BALB/cAnCrl, and NOD/SCID) for protein quantitation experiments, and from n = 3 male and n = 3 female C57BL/6NCrl mice for assay development experiments.
Field-collected samples	The study did not involve field-collected samples.
Ethics oversight	All sample processing performed at the University of Victoria - Genome British Columbia Proteomics Centre was performed under the approval granted by the University of Victoria Animal Care Committee. The TCP Animal Care Committee reviewed and approved all procedures conducted on animals at TCP and procedures were performed in compliance with the Animals for Research Act of Ontario and the Guidelines of the Canadian Council on Animal Care.

Note that full information on the approval of the study protocol must also be provided in the manuscript.
